# Comparison of T_2_ and T_2_^*^-weighted MR molecular imaging of a mouse model of glioma

**DOI:** 10.1186/1471-2342-13-20

**Published:** 2013-07-18

**Authors:** Barbara Blasiak, Samuel Barnes, Tadeusz Foniok, David Rushforth, John Matyas, Dragana Ponjevic, Wladyslaw P Weglarz, Randy Tyson, Umar Iqbal, Abedelnasser Abulrob, Garnette R Sutherland, Andre Obenaus, Boguslaw Tomanek

**Affiliations:** 1 Department of Clinical Neurosciences and Radiology, University of Calgary, 3330 Hospital Dr NW, Calgary, Alberta T2N 4N1, Canada; 2 Polish Academy of Sciences, Institute of Nuclear Physics, Krakow,152 Radzikowskiego, Krakow, Malopolska 31-342, Poland; 3 Thunder Bay Regional Research Institute, 980 Oliver Road, Thunder Bay, Ontario P7B 6V4, Canada; 4 Departments of Radiation Medicine, Radiology, Pediatrics, Loma Linda University Chan Shun Pavilion, Room A101011175 Campus Street, Loma Linda, California 92354, USA; 5 Faculty of Veterinary Medicine, University of Calgary, 3330 Hospital Dr NW, Calgary, Alberta T2N 4N1, Canada; 6 Department of Cellular and Molecular Medicine, Faculty of Medicine, University of Ottawa, 451 Smyth Road, Ottawa, Ontario K1H 8M5, Canada; 7 Human Health Therapeutics Portfolio, National Research Council of Canada, Ottawa K1A 0R6, Ontario Canada; 8 Alberta Innovates – Technology Futures, 3608 33 Street NW, Calgary T2L 2A6, Alberta, Canada

**Keywords:** Contrast-to-noise ratio, MRI, Molecular MRI, Contrast agents, Glioma

## Abstract

**Background:**

Standard MRI has been used for high-grade gliomas detection, albeit with limited success as it does not provide sufficient specificity and sensitivity to detect complex tumor structure. Therefore targeted contrast agents based on iron oxide, that shorten mostly T2 relaxation time, have been recently applied. However pulse sequences for molecular imaging in animal models of gliomas have not been yet fully studied. The aim of this study was therefore to compare contrast-to-noise ratio (CNR) and explain its origin using spin-echo (SE), gradient echo (GE), GE with flow compensation (GEFC) as well as susceptibility weighted imaging (SWI) in T2 and T2* contrast-enhanced molecular MRI of glioma.

**Methods:**

A mouse model was used. U87MGdEGFRvIII cells (U87MG), derived from a human tumor, were injected intracerebrally. A 9.4 T MRI system was used and MR imaging was performed on the 10 day after the inoculation of the tumor. The CNR was measured prior, 20 min, 2 hrs and 24 hrs post intravenous tail administration of glioma targeted paramagnetic nanoparticles (NPs) using SE, SWI, GE and GEFC pulse sequences.

**Results:**

The results showed significant differences in CNR among all pulse sequences prior injection. GEFC provided higher CNR post contrast agent injection when compared to GE and SE. Post injection CNR was the highest with SWI and significantly different from any other pulse sequence.

**Conclusions:**

Molecular MR imaging using targeted contrast agents can enhance the detection of glioma cells at 9.4 T if the optimal pulse sequence is used. Hence, the use of flow compensated pulse sequences, beside SWI, should to be considered in the molecular imaging studies.

## Background

MRI has been widely recognized as a diagnostic tool for early cancer detection, treatment monitoring and image guided surgery. Of particular interest is imaging of high-grade gliomas due to their rapid growth [1] and very poor prognosis with a median survival rate of only 9 months [2].

Standard contrast enhanced MRI, including application of Gd-based T_1_ contrast agents [3,4] do not provide sufficiently high specificity for tumor diagnosis and thus require targeted contrast agents which can be applied to provide information on tumor status (e.g. [5-7]). One approach to apply a molecular contrast agent which is usually composed of a superparamagnetic core and a shell of varying composition and size [8,9]. The superparamagnetic core reduces T_2_, T_2_*, and to a lesser degree, T_1_ relaxation times while the shell of the contrast agent is typically utilized to decrease toxicity and to allow nanoparticle (NP) functionalization [10]. Furthermore, increased core size increases T_2_ shortening and decreases T_2_/T_1_ ratio [11]. The small size of iron based NPs (usually 5–20 nm) and their strong impact on T_2_ and T_2_*, even in very small concentrations, make them ideal compounds for application to molecular imaging. Their T_2_ relaxivity can be up to 20 times that of Gd-DTPA [12].

While the impact of the size and composition of targeted contrast agents on MR properties have been studied using standard pulse sequences, optimization of pulse sequences for molecular imaging in animal models of gliomas have not been yet fully characterized. The competing requirements of molecular contrast imaging are to minimize cytotoxicity and maximize signal detection *in vivo*, and thus, require application of low concentrations of the contrast combined with an imaging technique that provides optimum contrast-to-noise ratio (CNR) enabling clinical application. While optimum pulse parameters for *ex vivo* experiments, when T_2_ and T_2_* are known, are relatively easy to establish, *in vivo* experiments are more complex as they include physiological parameters such as respiration, heart rate or blood flow that are very difficult to predict theoretically and to include into pulse sequence parameters. While there are methods for reducing motion artifacts, such as gating (e.g. [13]), data post processing [14] or ordered phase encoding [15] these methods do not address spin dephasing between excitation pulses and data acquisition due to fluid flows. To overcome these MR sequence shortfalls, we applied a pulse sequence that uses flow compensating gradients, known as gradient moment nulling (GMN) [16-18]. The goal of our studies was to optimize CNR using spin echo (SE), gradient echo (GE) and gradient echo with flow compensation (GEFC) in contrast-enhanced molecular MRI at 9.4 T. As susceptibility weighted imaging (SWI) [19,20] is frequently used for molecular MRI we also converted GE images into SWI as reference images. An *in vivo* model was used for evaluating CNR of antibody-targeted iron nanoparticles in transplanted glioma using a range of pulse sequences to assess the vascular density of the tumor.

## Materials and methods

### Tumor cell preparation

Details of the tumor and cell preparation have been previously published (e.g. [1]). Briefly, the U87MGdEGFRvIII cell line (U87MG) was derived from a human tumor known to express high levels of vascular endothelial growth factor and epidermal growth factor receptor [21]. This cell line was provided by the Ludwig Institute for Cancer Research (La Jolla, California, USA). The U87MG implants grow as solid, nonencapsulated spheroidal tumors. The tumor displays a dense vascular network, with many of the characteristics of glioblastoma vessels [3,7] including tortuous vessels with abnormal vascular basement membranes and increased permeability.

U87MG cells were cultured in DMEM solution supplemented with 10% fetal calf serum and maintained in a humidified 5% CO_2_ atmosphere at 37°C. Cells were harvested by trypsinization in ethylenediaminetetraacetic acid (EDTA)/trypsin, washed in phosphate-buffered saline (PBS), and centrifuged three times at 200 G. Viability was assessed using a 0.4% trypan blue exclusion test. After cell density was determined, cells were brought into suspension at a final concentration of 5 × 10^4^/2.5 μL and mixed with 2.5 μL of matrigel for a total volume of 5 μL. Cells were kept on ice until inoculation.

### Tumor model

Six CD-1 nude mice (male, 6 weeks old, Charles River, Canada) were anesthetized by intraperitoneal injection of a mixture of ketamine (8 mg/kg) and xylazine (6 mg/kg) and placed in a stereotactic head frame (Kopf Instruments, Tujunga, CA). Tumor cells were inoculated using procedures described previously [1,3,7]. Briefly, the scalp was shaved and swabbed with iodine and alcohol. The skin was incised and a 0.18 mm diameter hole was drilled in the skull. Approximately 5 × 10^4^ U87MGdEGFRvIII glioma cells, suspended in a total volume of 5 μL, were injected intracerebrally into the frontal lobe of each mouse with a chromatography syringe at a depth of 2.5-3 mm (1 mm anterior and 1.8 mm lateral to the bregma). Subsequently, the bony calvarium was sealed by a droplet of bone wax to prevent reflux and the skin was sutured. After the surgery, animals were allowed to recover from the anesthesia and were placed in their cages. All animal procedures were approved by the local Animal Care Committee.

### Contrast agent synthesis and injection

Commercially available iron oxide NPs were used (Nanotech-Ocean, USA). The NP consists of the mean core Fe_3_O_4_ diameter of 20 nm embedded in dextran matrix, with a hydrodynamic diameter of about 63 nm [21-23]. The NPs were functionalized with IGFBP7-sdAb [24,25], an antibody that binds with high specificity to glioma vasculature. Intravenous tail injection was used to deliver the contrast agent after the first series of MR images was obtained.

### Histology

**Figure 1:**
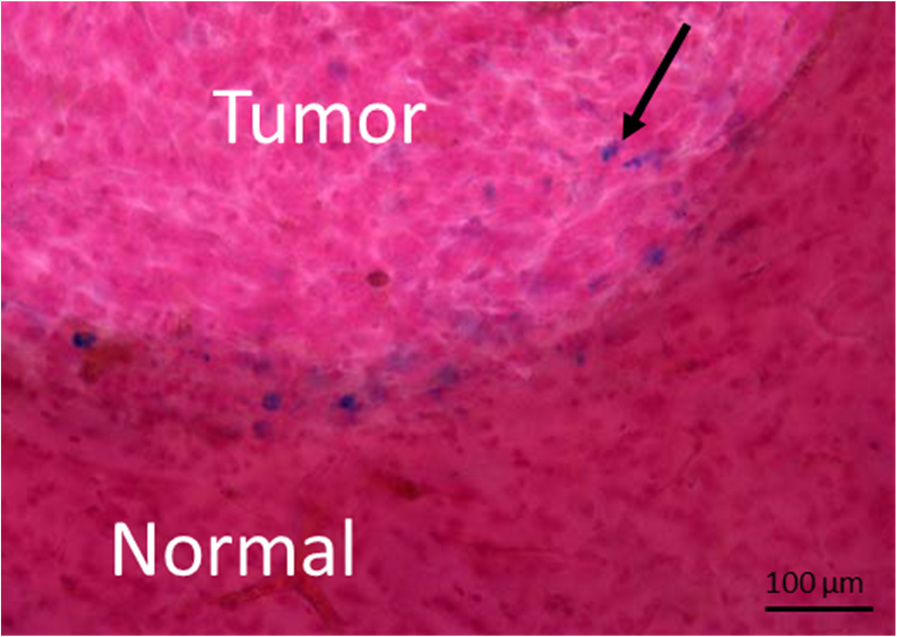
**Microscopic images of mouse glioma sections obtained using Prussian Blue staining collected after the last MRI session (24 hours after intravenous injection of the targeted contrast agent).** The image shows accumulation of the iron within the tumor (as indicated by the black arrow). Blue spots indicate iron, red - nuclei and pink - cytoplasm.

To confirm accumulation of the contrast within the tumor, histology was performed at the end of the experiment. Mice were sacrificed by intracardiac perfusion with heparinzed saline and their brains were excised and fixed in formalin (Figure 1). Coronal sections (50 μm) were obtained using a Vibratome (Ted Pella, Redding, California). Brain tissue sections were examined for the presence of iron nanoparticles by an Iron Stain Kit (Sigma) as per manufacturer’s instructions. Briefly, the sections were incubated for 30 min at room temperature with iron staining solution (a 1:1 mixture of 4% potassium ferrocyanide and 4% hydrochloric acid). Sections were then washed in deionized water and incubated for 3 min with 1% pararosaniline solution diluted 1/50 in water, followed by additional washing with deionized water. Tissue sections were then mounted on Superfrost Plus microscope slides (Fisher Scientific, Nepean, ON, Canada), cover slipped using mounting media and examined under a light microscope.

### MRI protocol

The MRI sessions started 10 days after cell inoculation when the tumor was about 2 mm in diameter. A 9.4 T/21 cm horizontal bore magnet (Magnex, UK) with a Biospec console (Bruker, Germany) was used. A volume (3 cm diameter, 2.5 cm long) radio-frequency coil was placed over the animal’s head covering the region of interest namely frontal cortices. For *in vivo* MRI experiments, a 2 mg Fe/ml concentration of the functionalized contrast agent was used [26] and 200 μl of the contrast agent was slowly (2–3 min) administered via tail vein using a 0.5-ml insulin syringe with a 27-gauge fixed needle (vehicle, 0.9% saline).

Three pulse sequences were tested: spin echo (SE), gradient echo (GE) and GE with flow compensation (GEFC). The MRI session started with SE 20 min after contrast injection (SE lasted 10 minutes) and it was followed by GE and GEFC CNR was calculated for each pulse sequence and eight echo times (TE) for each pulse sequence were tested to find the TE that provided maximum CNR. T_2_- and T_2_*-weighted axial images were acquired at the level of the tumor. FOV = 2 × 2 cm and slice thickness of 1 mm were used for each pulse sequence. For 2D GE we used the following parameters: TR = 50 ms, 10 continuous slices, 10 averages, 78 kHz bandwidth (BW), 1 ms Hermit selective pulse with a 15 degree flip angle, echo time (TE) 3, 7, 11, 15 and 19 ms. For 2D GEFC: TR = 50 ms, TE = 7 ms, 1 ms sin10h selective pulse with a flip angle 15°, BW = 50 kHz, 2 continuous slices, 10 averages were applied. A multiecho 2D SE sequence was used with TR = 5000 ms, 1 average, 10 continuous slices, 16 echoes, 10 ms apart each, first echo at 10 ms. Matrix size was 256 × 256 for SE and 128 × 128 for GE and GEFC. Total data acquisition time was 10 min for SE, 1 min for GE and 1 min for GEFC.

The SW images, for both GE and GEFC data, were processed as described by Haacke et al. [19]. The raw time-domain data were zero filled to 512 × 512 prior to 2D Fourier transformation and a phase image generated in the frequency domain. A high-pass filter was used to remove the low-spatial-frequency phase as follows: the central 48 × 48 points were used to create a phase image which was then used to subtract out the low-frequency phase components of the original 512 × 512 phase image. A mask was then calculated to multiply the 512 × 512 magnitude image using the following rule designed to enhance pixels of positive phase:

(1) $$\begin{array}{l} f\left(x,y\right)=\frac{\pi -\phi \left(x,y\right)}{\pi }\;\mathrm{for}\;\pi >\phi (x,y)>0\\ f\left(x,y\right)=1\;\mathrm{otherwise} \end{array}$$

This mask was multiplied with original magnitude image four times to produce the final SW image.

**Figure 2:**
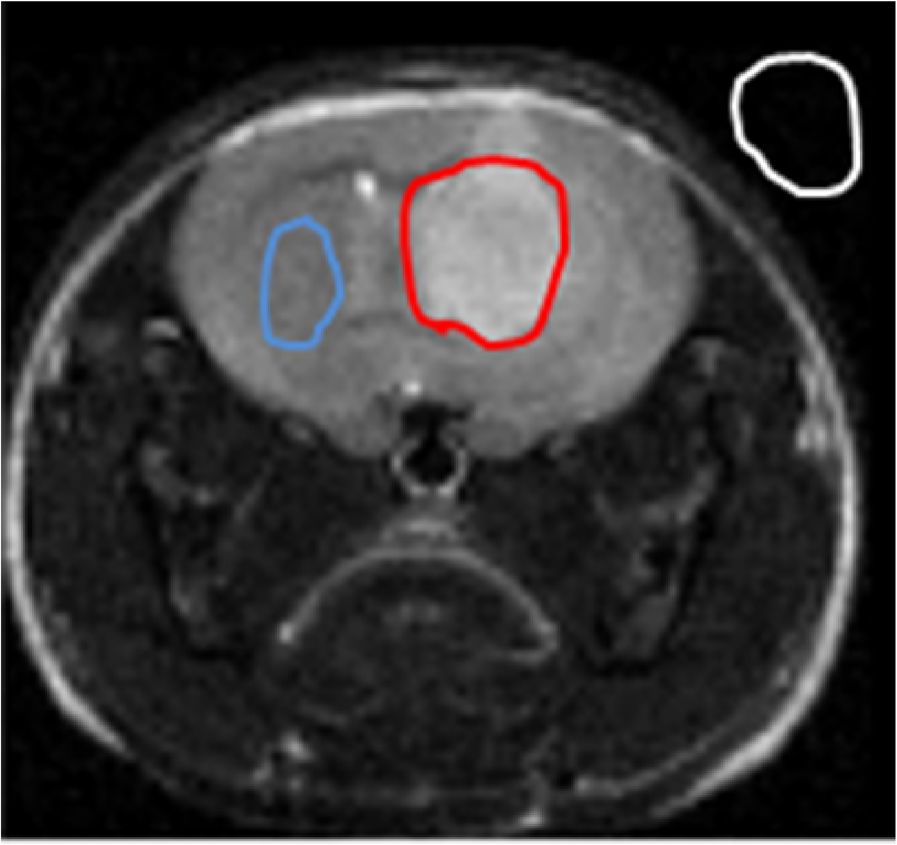
**A method of calculating CNR using ROIs. *SI(t) and SI(b) *are the averaged signal intensities within the tumor (red line) and a normal brain (blue line) region respectively; *Noise *was measured outside the mouse head (white line).** ROIs were selected using pre-contrast SE pulse sequence.

The applied GEFC pulse sequence uses first order flow compensation gradients in three directions. The flow compensation gradients reduce the signal loss due to flow. In our study TE = 7 ms was found to provide the maximum CNR thus that value was used for all scans. SNR and CNR were calculated as follows:

(2) $$\mathrm{SNR}\left(t\right)=\frac{\mathrm{SI}\left(t\right)}{\mathrm{Noise}}$$

(3) $$\mathrm{CNR}=\frac{\mathrm{SI}\left(t\right)-\mathrm{SI}\left(b\right)}{\mathrm{Noise}}$$

where *SI(t) and SI(b)* are the averaged signal intensities within the tumor and a normal brain region respectively; *Noise* is the averaged noise outside the rodent head (in the air) ROIs for tumor and brain were selected using pre-contrast SE pulse sequence, as areas within the tumor and the corresponding contralateral brain region (see Figure 2). Due to the different bandwidths (50 kHz vs 78 kHz) used for the GE and GEFC sequences which would result in a SNR advantage for GEFC all SNR and CNR values for GEFC were scaled by a factor of 0.8 (5078) to correct for this advantage.

## Results

**Figure 3:**
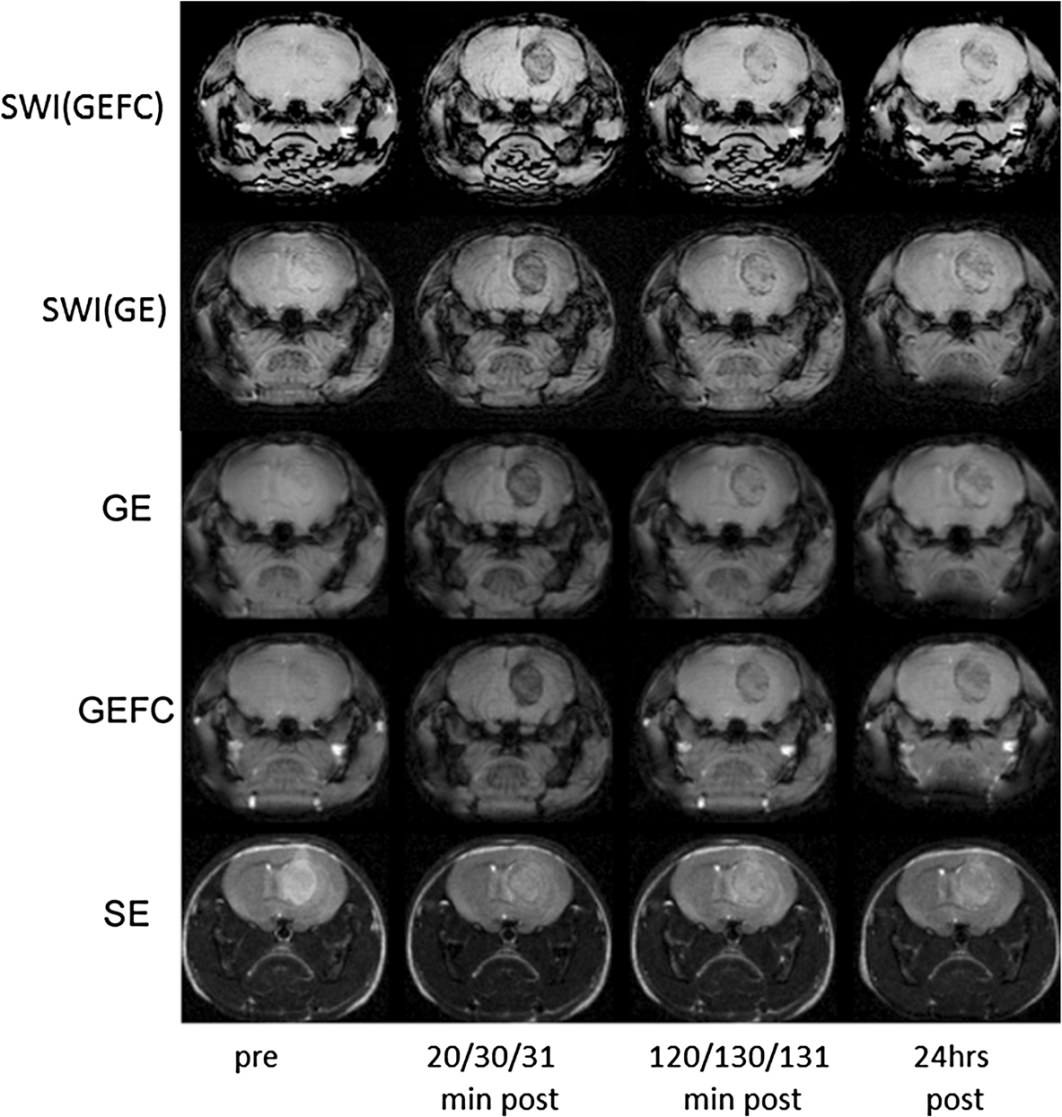
**MR images of the tumor bearing mouse using GE, GEFC, SE as well as SWI(GE) and SWI(GEFC) at the following time points after intravenous tail injection of targeted contrast agents: prior, 20, 120 min and 24 hrs post for SE; prior, 30, 130 min and 24 hrs for GE and prior, 31, 131 min and 2 hrs for GEFC.** TR/TE = 50/7 ms for GE and GEFC. TR/TE = 5000/60 ms for SE. FOV = 2 × 2 cm for each MRI. Note the increased negative contrast for GE and GEFC after contrast agent injection.

Several pilot experiments with different TEs for each pulse sequence were performed to optimize CNR. The optimum CNR was found to be TE = 7 ms for GE and GEFC pulse sequences and TE = 60 ms for SE. Examples of pre- and post-contrast MRIs using SE, GE and GEFC pulse sequences as well as SWI are presented in Figure 3. The pulse parameters remained unchanged for each MRI session. Pre-injection GE, GEFC and SW MR images showed very low contrast while pre-injection SE MRI showed good contrast between tumor and healthy brain tissues. The pre-injection CNR for each pulse sequence were significantly (p < 0.05) different from each other. Following contrast agent administration, CNR increased significantly for SWI, GE and GEFC pulse sequences, but decreased for SE. The absolute values of CNR for GE and SE were not significantly different at 20 min after injection, however the contrast was reversed: tumor was darker than normal tissue in GE and brighter in SE MRI.

**Figure 4:**
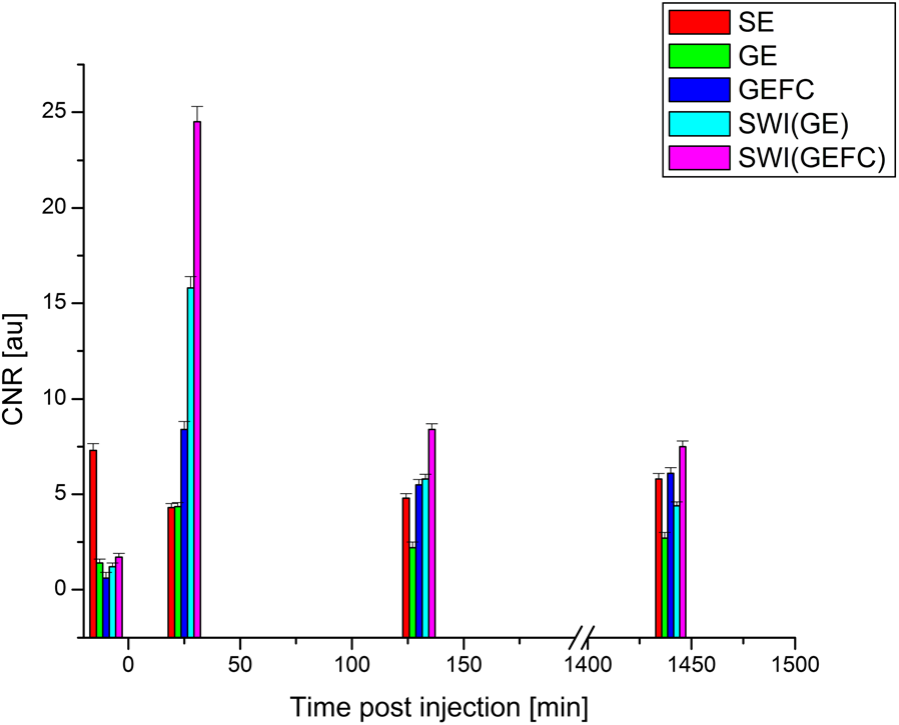
**Absolute CNR values for SE, GE, GEFC obtained prior, 20/30/31, 120/130/131 and 1440/1450/1451 min (~24 hrs) post intravenous tail contrast injection respectively.** SWI obtained from GE and GEFC data are also shown for comparison. Note the two-fold increase in CNR with the GEFC pulse sequence and even larger increase for SWI after injection of the targeted NP. (The sequence durations are not to scale for clarity.)

CNR was higher for GEFC 310 and 131 min after injection when compared to GE at 30 and 130 min, and SE at 20 and 120 min, but it was not significantly higher 24 hrs after injection when compared to SE. The changes in the absolute CNR values for each pulse sequence averaged over 6 animals are shown in Figure 4, while the corresponding CNR values are presented in Table 1. The contrast remained positive (tumor brighter) for SE and negative (tumor darker) for both GE and GEFC at each time point.

**Table 1:** **Comparison of CNR between the tumor and brain regions using GEFC, GE and SE pulse sequences as well as SWI at the respective time points: 20, 120 min and 24 hrs for SE; 30, 130 min and 24 hrs for GE; 31, 131 min and 24 hrs for GEFC post *iv *tail injection of the targeted contrast agent**

	**Pre**	**20/30/31 min**	**120/130/131 min**	**24 hrs**
GEFC	−0.6	−8.4	−5.5	−6.1
GE	1.4	−4.3	−2.2	−2.7
SE	7.3	4.3	4.8	5.8
SWI (GE)	−1.2	−15.8	−5.8	−4.4
SWI (GEFC)	−1.7	−24.5	−8.4	−6.9

The negative CNR value indicates that tumor is darker than normal brain.

In addition, based on multi-echo GE and single exponent echo train fitting, we measured T_2_^*^ of tumor and brain areas. T_2_^*^ within the tumor area decreased almost two fold (from about 15 ms to about 7 ms) following injection. Brain T_2_^*^ decreased by about 15% (from about 16 ms to 14 ms) in the same time. While the T_2_* of the blood could not be directly measured it was estimated based on the expected contrast agent concentration. We injected 0.4 mg Fe into the mouse with an approximate total blood volume of 1.9 ml resulting in an estimated iron concentration of 3.7 mM. The R_2_ relaxivity of the contrast agent is about 100 mM^-1^ s^-1^ at 9.4 T based on other agents with a similar core and hydrodynamic size [26]. Therefore, if the blood initially had a T_2_^*^ value of ~10 ms, just after administration of the contrast agent the T_2_^*^ would be about 2 ms and would gradually increase towards its initial value at the later time points.

There is a lack of signal from arteries in GEFC MRI 31 min after injection caused by the high concentration of iron oxide in the blood due to the extremely short T_2_^*^. Such a short relaxation time overwhelms the usual time-of-flight inflow enhancement seen in the arteries in the other flow compensated images. The brain and tumor signal-to-noise (SNR) before and at different time points after injection was about 10% higher for GEFC than for GE (Table 2) after correcting for the bandwidth difference.The SNR of the brain for SE decreased 20 min after injection by about 20%: from 34.3 to 27.8 for brain. The CNR (Table 1 and Figure 4) was almost twofold higher for GEFC 31 and 131 min after injection than for GE at 30 and 131 min, and 3.7 and 5.7 times higher after injection for SWI obtained from GE and GEFC respectively when compared to either the SE or GE sequences. The CNR decreased about 3 times for both SWI(GE) and SWI(GEFC) 130 min post injection and 4 times after 24 hrs yet remained about 2–3 times higher than GE or GEFC. This demonstrates that SWI based on GEFC indeed provides superior CNR for glioma detection when a targeted NP is utilized.

**Table 2:** **Comparison of SNR from the normal brain for SE, GE and GEFC pulse sequences pre, and at the respective time points: 20, 120 min and 24 hrs for SE; 30, 130 min and 24 hrs for GE; 31, 131 min and 24 hrs for GEFC post *iv *tail injection of the targeted contrast agent**

	**Pre**	**20/30/31 min**	**120/130/131 min**	**24 hrs**
SE	25.3	19.7	25.9	26.2
GE	29.0	18.7	24.5	25.4
GEFC	31.2	21.1	25.9	29.8

The standard deviation was smaller than ±1.3 for each measurement. The SNR for GE is corrected for the bandwidth difference (0.8).

## Discussion

Early detection of glioma, when the tumor is about a millimeter in size, may be associated with long-term survival [27,28]. However, conventional anatomical imaging techniques based on SE providing T_1_ and T_2_-weighted MRI typically can only detect neoplasias of several millimeters or larger, which contain approximately 1 million cells. Such large tumor size greatly decreases the odds of survival [28-31]. Furthermore, current clinical tumor segmentation methods require a trained operator’s input and is based on manual marking of tumor edges on T_2_-weighted MRI [29,30]. Therefore, early detection of the tumor and precise, accurate and fast determination of the tumor position and its boundaries are of particular clinical importance.

Application of SE pulse sequence allows tumor to be brighter before contrast application due to longer T_2_ tumor values. The elevated T_2_ values of high-grade gliomas involve many processes within tumor cells and their associated tumor blood vessels [29-32]. High-grade glioma angiogenesis results in hypervascularization, tortuous vessels exhibiting increased permeability, vasogenic edema, retention of plasma fluids and proteins within the extracellular space [1,3,7,33-37]. Furthermore, overexpression of CXCR4, a chemokine receptor known to mediate glioma cells invasiveness, has been correlated with increased T_2_[30]. All these factors contribute to a longer T_2_ value within the tumor compared to healthy brain tissues thus leading to tumor hyperintensity and high positive contrast in SE T_2_-weighted images. This is in contrast to GE based techniques which have very little innate contrast. GE, GEFC and SW MR images prior to injection of the NP in our study showed very low contrast between tumor and healthy brain tissues.

Application of a superparamagnetic contrast agent reduces T_2_ and T_2_* of both brain and tumor decreasing their SNR. It has been previously reported that the high intravascular blood volume and vessel leakage in glioma causes more contrast to be delivered and accumulate in the tumor than in the brain [3,38,39] decreasing the T_2_ and T_2_* of the tumor more than that of the brain [40,41]. This decreases the CNR in SE sequences (which start with positive contrast) and generates high negative contrast in GE sequences. This observation was verified with post-injection images demonstrating significantly increased absolute CNR for the GE, GEFC and SW(GE) and SW(GEFC) images (by 2.9, 7.8, 14.8 and 22.2 respectively), but decreased CNR for SE (by 3.0). It should also be noted, that acquisition time of SE is much longer than GE-based technique which makes SE less suitable for molecular imaging.

The best CNR was achieved with the SW images processed from the GEFC sequence at 30 min post injection, but similar contrast was obtained at the 131 min and 24 hour time point. This is expected as SWI is known to be very sensitive to superparamagnetic iron based contrast agents [19,20]. The GEFC images also showed better contrast compared to the GE images. This result is likely caused by at least two phenomena, that each contributes to the overall significant differences between GE and GEFC. We partially attribute the results to an increased SNR in the GEFC images due to the action of the flow compensation gradients and to an increased cancelation between signals from the blood and brain tissues. The increase of SNR in GEFC depends mostly on the efficacy of the applied compensating gradients, namely their proper balancing. The signal cancelation from the brain and blood should be considered in the terms of contrast agents containing superparamagnetic NPs that cause a local susceptibility effect leading to a phase shift within the blood vessels [42,43]. This phase shift can cause the signal from the blood to cancel with the signal from the tissue at appropriate echo times [42-47]. The increased signal intensity of the blood vessels due to the flow compensation may enhance this signal cancelation leading to lower signal intensities in voxels that contain both tissue and blood vessels. However, the estimated very short T_2_* value (~2 ms) of blood at the 30 min time point would virtually eliminate any signal from the blood and therefore prevent any significant cancelation at this time point. While some cancelation could occur at later time points, the GEFC shows higher CNR at all time-points, which makes signal cancelation unlikely to be the main cause for the improved CNR. Likewise, measured SNR values only showed a difference of about 10% between GE and GEFC after correction due to the different receiver bandwidths. The CNR changes observed were significantly larger than 10% and cannot be wholly attributed to a simple increase in the SNR of the GEFC scan.

It should be also noted, that magnetic susceptibility difference between blood vessels and surrounding tissue impacts signal of both GE and GEFC. For vessels that are not parallel to the main magnetic field (B_0_), the susceptibility difference creates extravascular field inhomogeneities, thus strong T_2_^*^ decrease independent of the blood flow. Considering isotropic distribution of tumor vessels’ orientation and preferred direction introduced by B_0_, a substantial fraction of the vessels is oriented at angles larger than 50° with respect to the main magnetic field [48]. The field inhomogeneities at larger angles reach far beyond the actual vessel [47,49] thus the flow compensation is more efficient enhancing contrast due to the signal cancelation in the direction parallel rather than perpendicular to the main magnetic field diminishing overall contrast improvement that would be expected solely from the cancellation between blood and surrounding tissue. As seen above there are various potential mechanisms that may be responsible for the improved CNR for GEFC when compared to GE. However neither of these putative mechanisms can sufficiently explain the observed changes. While the above discussion provides some explanation further studies into the origin of these changes are warranted.

We presume that the decrease in CNR for both GE and GEFC (with a post-injection time of 2 and 24 hrs) but sustaining CNR above pre-injection levels, could be due to preferential retention of superparamagnetic NPs within the tumor compared to the normal brain tissue due to selective immunoaffinity of the targeted contrast agent. These observations are important not only for glioma detection within an experimental setting, but are also applicable for clinical diagnosis. The results of these studies with targeted superparamagnetic contrast agents suggest that the best CNR is provided by SWI(GEFC) compared to SE and GE pulse sequences. It should be however noted that GE based pulse sequences and hence SWI are prone to artifacts, in areas such as auditory canal or frontal lobes, due to their sensitivity to susceptibility effects. Our results also demonstrate that molecular MR imaging using targeted contrast agents can enhance the detection of a relatively small number of glioma cells if an improved and optimal pulse sequence is used. Of particular interest is also the fact that CNR is higher just after injection and remains higher at 24 hrs point for GEFC and thus SWI(GEFC). This is important for imaging comparing non-targeted and targeted iron based contrast agents. The first time point (~20-30 min) is important as then non selective accumulation occurs. At the last time point (~24 hrs) targeted contrast agent accumulation can be observed as more NPs are expected to selectively bind to the tumor unlike non-target NPs that are washed out by that time. Thus the results could also be used for improved differentiation between targeted and non-targeted contrast agents at diagnostically important time points.

## Conclusion

The appropriate use of SWI and flow compensated pulse sequences needs to be considered in the ongoing development of molecular imaging, particularly in vasculature rich tissues.

## Competing interest

The authors declare that they have no competing interests.

## Authors’ contributions

BB designed the study, performed MRI experiments, statistical data analysis and prepared manuscript; SB, AO analyzed SWI data, provided explanation of the results; TF optimized pulse sequences, obtained preliminary data; DR performed cell inoculation; WPW, GS provided input on the experimental design and data interpretation; JM, DP performed cell culture and tumor model; RT performed SWI; UI, AA provided histology and synthetized contrast agents; BT conceived the idea and participated in data analysis, supervised the study; all authors revised the article critically, read and approved the final manuscript.

## Pre-publication history

The pre-publication history for this paper can be accessed here:

http://www.biomedcentral.com/1471-2342/13/20/prepub
